# Lower extremity physical function and quality of life in patients with stroke: a longitudinal cohort study

**DOI:** 10.1007/s11136-024-03713-0

**Published:** 2024-06-25

**Authors:** Michelle Ryan, Roland Rössler, Nikki Rommers, Laura Iendra, Eva-Maria Peters, Reto W. Kressig, Arno Schmidt-Trucksäss, Stefan T. Engelter, Nils Peters, Timo Hinrichs

**Affiliations:** 1 University Department of Geriatric Medicine Felix Platter, University of Basel, Basel, Switzerland; 2 Department of Clinical Research, University of Basel, University Hospital Basel, Basel, Switzerland; 3 Department of Neurology and Stroke Center, University Hospital Basel and University of Basel, Basel, Switzerland; 4 Division of Sport and Exercise Medicine, Department of Sport, Exercise, and Health, University of Basel, Grosse Allee 6, Basel, 4052 Switzerland; 5 Neurology and Stroke Center, Klinik Hirslanden, Zurich, Switzerland

**Keywords:** Quality of life, Stroke, Rehabilitation, Timed up and go test, Mobility limitation, Physical function

## Abstract

**Purpose:**

Lower extremity physical function (LEPF) is a key component for mobility and is impacted in stroke-related disability. A reduction in LEPF can have a significant impact on an individual’s Quality of Life (QoL). The aim of this study is to characterise the relationship between LEPF and QoL.

**Methods:**

The MOBITEC-Stroke Study is a longitudinal cohort-study including patients with their first occurrence of ischaemic stroke. Using a linear mixed-effects model, the relationship between LEPF (timed up-and-go performance (TUG); predictor) and QoL (Stroke Specific Quality of Life scale (SS-QoL); outcome) at 3 and 12 months post stroke was investigated and adjusted for sex, age, Instrumental Activities of Daily Living (IADL), fear of falling (Falls Efficacy Scale-International Version, FES-I), and stroke severity (National Institute of Stroke Severity scale, NIHSS), accounting for the repeated measurements.

**Results:**

Data of 51 patients (65 % males, 35% females) were analysed. The mean age was 71.1 (SD 10.4) years, median NIHSS score was 2.0. SS-QoL was 201.5 (SD 20.5) at 3 months and 204.2 (SD 17.4) at 12 months; the mean change was 2.7 (95% CI -2.4 to 7.7), p= 0.293. A positive association was found between baseline TUG performance (estimate log score -13.923; 95% CI -27.495 to -0.351; p=0.048) and change in SS-QoL score in multivariate regression analysis.

**Conclusion:**

Higher LEPF (i.e better TUG performance) at baseline, was associated with an improvement in QoL from 3- to 12-months post stroke. These results highlight the critical role of physical function, particularly baseline LEPF, in influencing the QoL of stroke survivors.

## Abstract (summary)

Lower extremity physical function (LEPF), which is a measure of mobility, affects the quality of life in people who have had a stroke. Understanding this relationship can help improve stroke survivors’ recovery and well-being.

This study sought to understand the connection between mobility, specifically lower extremity physical function, and the quality of life in individuals after a stroke.

The study’s primary focus is to explore and characterize how LEPF relates to the quality of life in stroke patients over time, particularly between 3- and 12-months post-stroke.

It was found that better physical function (measured by the timed up-and-go test) at the beginning of the study was linked to improved quality of life after 12 months. This suggests that early improvement in mobility, particularly lower extremity physical function can have a positive impact on the overall quality of life for stroke survivors.

## Introduction

Stroke is a major cause of disability worldwide [1] and has been demonstrated to have a significant impact on quality of life (QoL). In 2019, 143 million disability-adjusted life years (DALYs) were associated with stroke [1] and stroke-related disability is expected to increase in line with higher prevalence and improved stroke survival rates [2, 3]. The 5-year cumulative stroke recurrence risk is deemed at 26.4% [4] while the global stroke incidence has increased by 70% and its prevalence by 85% [1, 5].

Stroke has a major impact on mobility [6] and one such measure of mobility is lower extremity physical function (LEPF) [7]. Stroke rehabilitation aimed at enhancing independence in daily activities and specific lower extremity functional abilities such as balance, gait function [8] and gait speed [9] have been found to improve QoL. It is vital that patients maintain adequate mobility and physical activity levels as a non-pharmacological component (lifestyle modification) of secondary stroke prevention [10] as well as reducing the fear of falling and subsequent falls [11]. The participation in a post-stroke cardiac rehabilitation recovery program with the inclusion of physical activity has been demonstrated to improve overall function and all-cause mortality [12].

Despite these positive findings regarding mobility and functional outcomes, there is still uncertainty regarding the long-term development of functional mobility including LEPF and QoL post stroke [7, 9]. As a result, there is an urgent need to characterise the factors that are associated with QoL following stroke, including the temporal relationship between lower extremity physical function (LEPF) and QoL. This is in keeping with wider research that emphasises the importance of QoL as a key priority for patients in their post-stroke recovery [13]. Additional research is required to gain a deeper understanding of the relationship between LEPF and QoL in stroke survivors.

The aim of this study is to characterise the relationship between LEPF and QoL of patients from three to twelve months following an ischaemic stroke. It was hypothesed that there is an association between the change scores (i.e. from 3 to 12 months post stroke) in LEPF (as measured by TUG) and QoL.

## Methods

### Study design

Data was obtained from the MOBITEC-STROKE project (ISRCTN85999967), a prospective observational cohort study (*N* = 59 patients) conducted in Basel, Switzerland [7]. Participants were assessed at four time points: at 3 (baseline assessment), 6-, 9- and 12-months post stroke. QoL was assessed at 3- and 12-months post stroke and therefore data collected at these two time points were used for the present analyses. The study was approved by the Ethics Committee of Northwestern and Central Switzerland (Reg.-No. 2019 − 00989). All participants provided written informed consent. Further details can be reviewed in the study protocol [7]. This is a secondary analysis and the study was not specifically powered to perform this statistical analysis.

In designing and reporting this study, we adhered to the Strengthening the Reporting of Observational studies in Epidemiology (STROBE) guidelines to ensure comprehensive and transparent reporting of our research findings [14].

### Study population

MOBITEC-Stroke included community-residing ambulatory patients, who experienced their first ischaemic stroke. Inclusion criteria were as follows: first occurrence of ischaemic stroke within the previous 3 months, age ≥ 18 years, ability to communicate verbally, full capacity to provide written informed consent to the study, ability to independently mobilise from a chair and to independently walk for 20 m (with or without a walking aid) at their own pace, however, without assistance from another individual. The presence of at least one of the following stroke-related symptoms potentially impacting mobility also had to be present to be included in the study: lower limb paresis, stance/gait ataxia, visual field defect or visual disturbance, central vestibular dysfunction, or attentional deficit/neglect. Exclusion criteria were as follows: nursing home or assisted living resident; inability to walk unaided (modified Rankin Scale, mRS, > 3); severe cognitive deficit (Montreal Cognitive Assessment score < 21 or, for persons with ≤ 12 years of education, < 20); terminal illness, orthopaedic surgery of lower limbs within the previous year/recipient of rehabilitation following a surgical procedure at the time of stroke occurrence, acute psychiatric illness including depression; patients with major difficulties walking or climbing stairs pre-stroke were excluded from the study.

Patients who presented to the Stroke Centre, University Hospital Basel with an acute ischaemic stroke between October 2019 and March 2021 were screened for eligibility. Patients who were eligible were offered participation in the study. Cessation of recruitment occurred when the target sample size of *N* = 59 was reached. Further information regarding the establishment of the study sample size can be reviewed in the study protocol [7].

### Measures

#### Outcome

The outcome of interest was the association between the change in Stroke Specific Quality of Life (SS-QoL) score, and LEPF over time. SS-QoL consists of 49 items covering 12 aspects of post-stroke quality of life [15, 16]. Scores range from 45 to 245, with higher scores indicative of higher QoL. These aspects include energy, family roles, language, mobility, personality, self-care, social roles, thinking, upper extremity function, vision, and work/productivity [17]. The SS-QoL’s psychometric properties have been validated in patients who have experienced ischemic stroke and intracerebral haemorrhage [15, 18, 19]. This self-report questionnaire demonstrates excellent content validity, as it was developed in collaboration with post-stroke patients who defined the scale’s various domains. Construct validity as a measure of Health-Related Quality of Life (HRQoL) has also been established [16]. The total SS-QoL score has been validated for German speakers, however certain subscales including energy, mood and thinking have not been validated and further research is required [15]. Internal consistency has been deemed to range from adequate to excellent with a Cronbach’s alpha value of 0.96 [16] while both test-retest reliability and inter-rater reliability were also demonstrated to be excellent [16].

### Exposure of interest

The timed up-and-go test (TUG) [20] is an accessible measure which can provide an assessment of general LEPF. In the TUG, participants rise from sitting to standing and then walk around a cone at a 3-meter distance. Walking aid and armrest use were permissible. The outcome measure of the TUG is the time taken in seconds from the command “Go” until the first seated contact with the chair. Lower TUG values indicate a better LEPF. The test was conducted by trained assessors under standardised laboratory conditions [7].

### Participant characteristics

The following sociodemographic parameters were assessed by self-report: living alone (“no” vs. “with at least one other person”), school education (years), and financial hardship (degree to which financial difficulties restricted everyday life over the past four weeks: “had no influence” vs. “has somewhat complicated my life” vs. “has significantly complicated my life”).

### Further independent variables

#### Age and sex

Demographic information regarding age and sex of participants were retrieved from clinical records.

#### Instrumental Activities of Daily Living (IADL)

Participants’ self-reported functional status in relation to Instrumental Activities of Daily Living (IADL) (i.e. difficulties in performing the respective task) was assessed using this 8-item questionnaire including the following functions: using the telephone, shopping, preparing food, housekeeping, doing laundry, using transportation, handling medication, and handling finances. A score of 1 was indicative of no difficulties, a score of 2 corresponded to light difficulties, a score of 3 was indicative of severe difficulties and a score of 4 indicated that it was not possible to carry out the respective activity [21]. A sum score for the 8 items was calculated (possible range 8 to 32).

#### Falls Efficacy Scale-International Version (FES-I)

The Falls Efficacy Scale-International Version (FES-I) is a self administered questionaire to assess the confidence level of individuals to complete daily activities without falling [22]. It is a 10 item scale whereby each score is rated on a scale of 1–10. A score of 100 indicates no confidence whereas a score of 1 indicates confidence. A score of ≥ 70 out of 100 indicates that an individual has a fear of falling [22, 23].

#### National Institutes of Health Stroke Scale (NIHSS)

National Institutes of Health Stroke Scale (NIHSS) is a 15-item scale that evaluates the neurological outcome and recovery of patients following a stroke. The scale assesses level of consciousness, extraocular movements, visual fields, facial muscle function, extremity strength, sensory function, coordination (ataxia), language (aphasia), speech (dysarthria), and hemi inattention (neglect) [24, 25]. Each item is scored from 0 to 2, 0–3 or 0–4. A score of 0 indicates no neurological impairment, a score of 1 to 5 mild, a score of 5 to 14 mild to moderately severe, and a score > 25 very severe neurological impairment. The maximum score is 42. The scale has been validated for the German language [24, 26].

#### The modified Rankin Scale (mRS)

The modified Rankin Scale (mRS) is a single item scale that is used to categorise the level of functional independence post stroke [27]. The scale is scored from 0 (no symptoms) to 6 (death) and has also been validated for German-speaking populations [26].

### Statistical analysis

#### Descriptive statistics

Demographic characteristics were summarized using descriptive statistics. Normally distributed variables were presented as mean (standard deviation), while non-normally distributed variables were reported as median (interquartile range).

#### Multivariate longitudinal data analysis

The relationship between LEPF (as measured by TUG performance at baseline (3 months post stroke) and change in TUG from 3 months to 12 months post stroke) and change in SS-QoL was investigated. The model was adjusted for a set of pre-defined covariates that showed univariable associations with the outcome and are considered clinical prognostic factors: age category (i.e. split by median), sex, IADL score, FES score, and NIHSS score. Additionally, we adjusted the model for baseline SS-QoL score. Due to the skewed distribution, TUG was log-transformed for these analyses. Furthermore, we assessed the pairwise correlations between the numeric variables to check for multicollinearity. A linear regression model was employed with change in SS-QoL score as the outcome, LEPF-variables as fixed effects of interest, and the above-described covariates as fixed effects that were not to be interpreted. The variance inflation factor was assessed for all variables in the multivariate model to rule out any multicollinearity, and further model assumptions were checked (e.g. normal distribution of the residuals). Model estimations, including coefficients, standard errors, and p-values, were derived. All analyses were performed in R version 4.2.2.

## Results

**Fig. 1:**
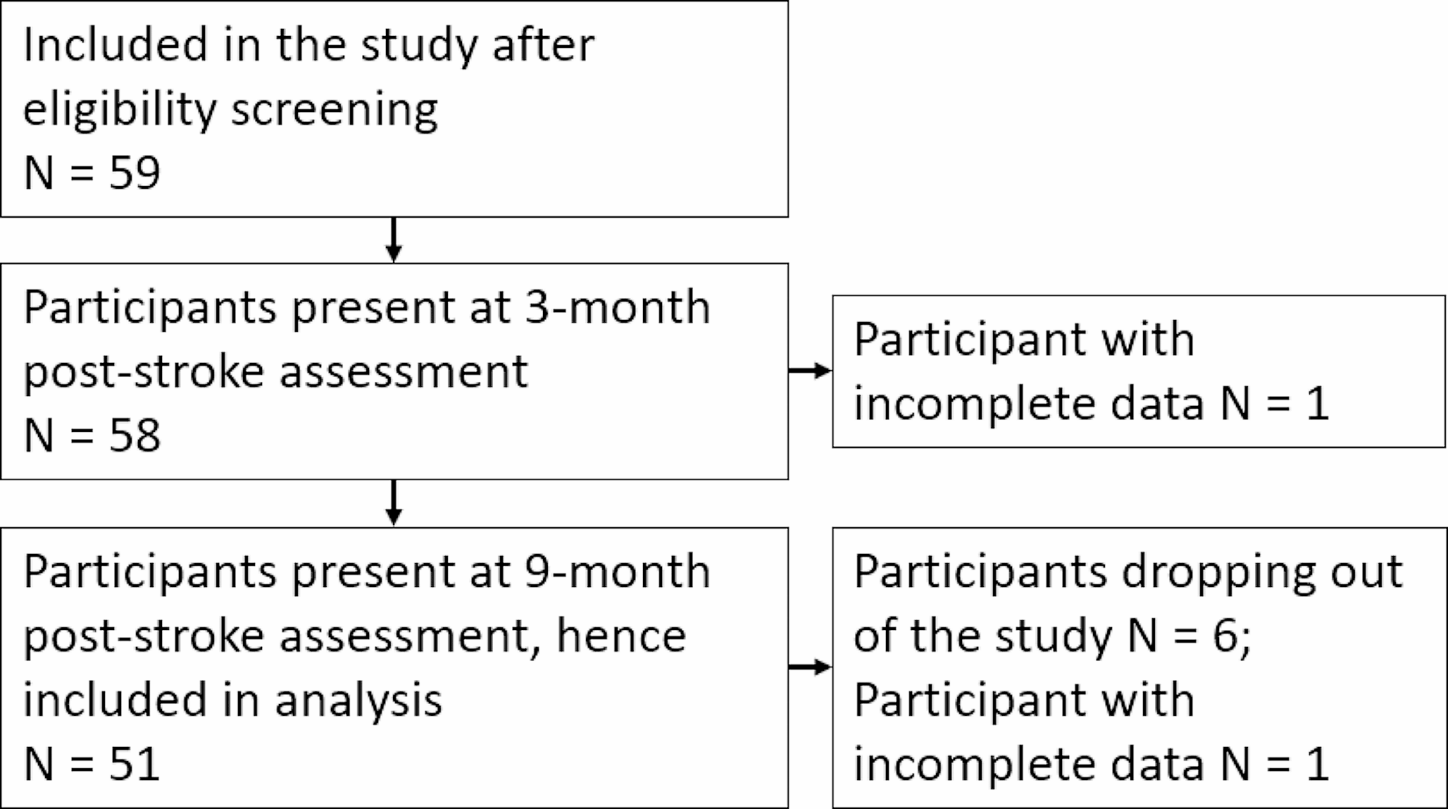
Flow of participants through the study

Out of 59 patients enrolled in the study, six participants dropped out of the study between baseline (at 3 months) and 12 months. In total, six participants dropped out due to health-related reasons (*n* = 3), fear of Covid-19 infection (*n* = 1), lack of interest (*n* = 2); another two participants had missing SS-QoL values. Accordingly, data of 51 community-residing ambulatory patients (33 males and 18 females) who experienced their first ischaemic stroke were analysed. Participant characteristics at baseline (3 months post stroke) are provided in Table 1. For further information regarding participant flow, please refer to a recent publication from the MOBITEC-Stroke project [28]. For the flow of participants through the study see Fig. 1.

**Table 1:** Baseline-characteristics of the study participants (i.e. 3 months post stroke)

Variable	Value
Number of Participants (N)	51
Sex = male (N, (%))	33 (64.7)
Age (mean (SD))	71.10 (10.32)
Living alone (N, (%))	27 (52.9)
Education, years (mean (SD))	13.45 (3.41)
Influence of Finances on Life (N, (%))	
“Had no influence.”	46 (90.2)
“Has somewhat complicated my life.”	4 (7.8)
“Has significantly complicated my life.”	1 (2.0)
NIHSS Scale [0–42, lower is better] (median [IQR])	2.0 [1.0]
mRS [0–6, lower is better] (median [IQR])	1.0 [1.0]
IADL [8–32, lower is better] (median [IQR])	10.00 [3.50]
FES-I [16–64, lower is better] (mean (SD))	20.88 (5.91)

*N* number of participants, *SD* standard deviation, *IQR* interquartile range, *NIHSS* National Institutes of Health Stroke Scale, *mRS* modified Rankin Scale, *IADL* Instrumental Activities of Daily Living, *FES-I* Falls Efficacy Scale-International Version

Descriptions of LEPF and QoL at 3 and 12 months after stroke are shown in Table 2. A mean increase of SS-QoL score of 2.7 points (95% CI -2.7 to 7.7) was observed between 3 months and 12 months post stroke. Similarly, a mean increase in TUG performance (-1.3 [95% CI -2.3 to -0.4] sec) was observed (see Table 2).

**Table 2:** Timed up-and-go (TUG) performance and Stroke Specific Quality of Life (SS-QoL) over time (*N* = 51)

		3 months		12 months			Change (i.e. 12 months – 3 months)	
Variable		Mean (SD)		Mean (SD)		Mean (SD)	95%CI	*p*-value
SS-QoL score		201.5 (20.5)		204.2 (17.4)		2.7 (17.8)	-2.4 to 7.7	0.293
TUG (sec)		10.1 (4.4)		8.7 (2.9)		-1.3 (3.4)	-2.3 to -0.4	0.006

*SS-QoL* Stroke Specific Quality of Life Scale, *SD* standard deviation, *TUG* timed up-and-go test; *CI* confidence interval

The multivariate regression model indicated a significant negative association between TUG time (log score) at baseline and development of SS-QoL (estimate: -13.923, *p* = 0.048), indicating that a longer time taken for the TUG test was associated with a decline in QoL. The results of the multivariate regression model are summarized in Table 3.

**Table 3:** Results of multivariate regression model for the analysis of the SS-QoL Change score from 3 to 12 months post stroke (*N* = 51)

Variable	Estimate	95%-CI lower	95%-CI upper	*p*-value
TUG Time Baseline (log score)	-13.923	-27.495	-0.351	0.048
TUG Time Change (log score)	0.558	-15.742	16.858	0.874

*TUG* timed up-and-go test; model was adjusted for age, sex, difficulties in performing Instrumental Activities of Daily Living (IADL score), falls efficacy (FES-I score), and stroke severity (NIHSS score). Model R² = 0.658, Cohen’s f² = 1.93

## Discussion

### Main findings and interpretation

The analysis adjusted for covariates demonstrated an association between LEPF and QoL. Notably, the effect size for our model, as indicated by Cohen’s f², is 1.93. This value significantly exceeds the threshold for a large effect size (f² = 0.35), suggesting a strong association between TUG scores and QoL changes. This underscores the importance of TUG performance as a predictor of QoL outcomes in individuals post-stroke, affirming its relevance in clinical assessments and interventions aimed at improving quality of life.

Importantly, for each additional second required to complete the TUG test at baseline (i.e. 3 months post stroke), we observed an estimated decrease of 1.37 points in SS-QoL scores. This finding is particularly noteworthy in the context of the minimal clinically important difference (MCID) for SS-QoL, which has been reported to be 4.7 points among patients with aneurysmal subarachnoid hemorrhage [29]. Our results suggest that even modest increases in TUG times – reflecting decreased mobility – could contribute to clinically meaningful deteriorations in quality of life. Given that a change of approximately 3.43 s in TUG time would approach the MCID for SS-QoL, our study underscores the critical importance of physical function in the recovery and rehabilitation of stroke survivors. These findings highlight the potential value of interventions aimed at enhancing mobility, such as targeted physical therapy, in significantly impacting patients’ quality of life post-stroke. Thus, incorporating regular assessments of physical function, using tools like the TUG test, into clinical practice could provide valuable insights into patients’ rehabilitation progress and overall well-being.

### Comparison with previous studies

A paucity of research exists regarding TUG being a predictor of QoL in Stroke patients. However, in other patient populations, higher measures of LEPF, including a quicker TUG performance and walking speed, have been demonstrated to be associated with higher values in QoL [30–32]. These studies, which are discussed in more depth below, included patients with Parkinson`s disease [32, 33] osteoporosis [30, 34] and musculoskeletal disorders [31].

Stegmöller and colleagues investigated the relationship between TUG performance and predictors of QoL on the Parkinson’s Disease Questionnaire (PDQ-39) for 1964 patients with a mean TUG score of 11.0 s [32]. Significant correlations were reported between TUG and each of the PDQ-39 domains. Ellis and colleagues reported a similar correlation between higher physical function performance and PDQ-39 domains for 263 patients with Parkinson`s disease, with a mean TUG performance of 13.2 s [33]. In a study describing an association between QoL, walking speed and TUG in patients with osteoporosis-related fractures, aged between 60 and 93 years (*n* = 155), participants were categorized in the “Fast” group if they demonstrated a mean TUG of 9.9 s [30]. A lower TUG performance was found to be associated with a lower health-related QoL [30]. Similarly, Hirano concluded that the TUG test may be a useful clinical tool to evaluate the QoL of patients with musculoskeletal disorders (*n* = 386). The mean TUG performance in their sample was 6.8 s [31]. The participants included in our study demonstrated a mean TUG performance of 10.1 (SD 4.4) seconds 3 months after the stroke and 8.7 (SD 2.9) seconds 12 months after the stroke, suggesting that the included patients had a relatively adequate physical function post stroke. The relatively high physical function of patients in this sample may be related to the study exclusion criteria which excluded patients with severe mobility restrictions and therefore patients who were included had generally mild symptoms post stroke. The median NIHSS score of 2 of the current study population is indicative of minor stroke severity which would also have an impact on general physical function. However, as the studies discussed above were cross-sectional in design, these results should be interpreted with caution.

Further research is required to investigate the role of the TUG as a tool not only to assess physical function and mobility as such but also its (predictive) value regarding broader aspects of meaningful recovery for patients following a stroke such as QoL and other patient-reported outcomes.

Improvements in other measures of LEPF including balance (Berg Balance Scale (BBS)), gait speed, and step length (non-paretic and paretic limb) [8] have been shown to be correlated with improvements in SS-QoL. Chen and colleagues demonstrated that the BBS was a major predictor of mobility and subsequent participation/role domains of the Health-Related Quality of Life (HRQoL) [35]. Gait speed is commonly used to quantify functional capacity of stroke patients. Improved QoL scores correlated with improved walking speed at 6-month follow-up [36]. Muscle strength and tone were also demonstrated to create an improvement in gait speed alongside balance [37] and therefore are deemed vital areas of focus in neurorehabilitation post stroke to improve patient QoL. The TUG can be considered a combined assessment of dynamic balance, lower extremity strength, and walking ability. As such, our findings are in line with the results reported in the above studies. Overall, the results of this study are supported by the findings of previous studies regarding the integration of improving LEPF in post-stroke rehabilitation and its relationship with QoL.

### Relevance for future interventions

LEPF has a positive impact on the self-reported QoL of individuals, as evident from the existing body of literature and the results of our study. Therefore, a quick recovery of physical function post stroke is relevant to further support the positive development of patients’ QoL.

### Strengths and limitations

Strengths of this study include the longitudinal design (i.e. repeated measures at specific time points) as well as the rigorously standardised laboratory conditions in our hospital. This study was limited by a relatively small sample size which impacts the generalisability of the study results. The sample size also limited the number of potential confounders to be included in the regression model. As the study was conducted during the Covid-19 pandemic, opportunities for participation in social activities were limited and thus these social restrictions may have had an impact on QoL and thereby the study results. The exclusion criteria of this study ruled out participants who were experiencing an acute depression and those unable to walk with personal assistance and therefore the results may not be representative for all patient groups following a stroke. Specific data on the duration of hospitalisation post-stroke and the precise vasculature affected were not collected. Future studies might consider collecting these data.

## Conclusion

The observed positive association between TUG performance at baseline and the development of SS-QoL over time underscores the importance of LEPF in influencing the QoL of stroke survivors. Additionally, it reinforces the value of TUG as a clinically relevant and practically easy-to-use assessment of LEPF in post-stroke patients.

These findings contribute to the existing literature by highlighting the critical role of physical function, particularly LEPF, in influencing the QoL of stroke survivors. However, it is essential to consider the multifactorial nature of QoL, and while LEPF is a significant determinant, other factors, both physical and psychosocial, may also play pivotal roles in shaping the overall QoL of stroke patients.

Further longitudinal studies and intervention studies are required to better understand the causal relationship between TUG performance and its predictive value for QoL. Enhancement of QoL can be considered a significant – if not the most important – determinant in the recovery of stroke patients. Identification and classification of the specific factors that are associated with improvements in QoL can facilitate the creation of tailored rehabilitation programmes that are centred on the needs of individual patients.

## Data Availability

The data that support the findings of this study are available from the corresponding author on reasonable request.

## Declarations

### Guarantor

As the study PIs, TH and NP are the guarantors of this research project.

## References

[1] Feigin, V. L., et al. (2022, January 1). World Stroke Organization (WSO): Global stroke fact sheet 2022. *International Journal of Stroke*, *17*(1), 18–29. 10.1177/17474930211065917/FORMAT/EPUB. 10.1177/17474930211065917 34986727

[2] Donkor, E. S. (2018). Stroke in the 21st century: A snapshot of the burden, epidemiology, and quality of life, *Stroke Research and Treatment*, *2018*. 10.1155/2018/3238165. 10.1155/2018/3238165 PMC6288566 30598741

[3] Grefkes, C., & Fink, G. R. (2020). Recovery from stroke: Current concepts and future perspectives. *Neurol Res Pract*, *2*, 17. 10.1186/s42466-020-00060-6. 2020/06/16. 33324923 10.1186/s42466-020-00060-6 PMC7650109

[4] Mohan, K. M., Wolfe, C. D. A., Rudd, A. G., Heuschmann, P. U., Kolominsky-Rabas, P. L., & Grieve, A. P. (2011, May). Risk and cumulative risk of stroke recurrence, *Stroke*, *42*(5), 1489–1494. 10.1161/STROKEAHA.110.602615. 10.1161/STROKEAHA.110.602615 21454819

[5] Owolabi, M. O., et al. (2022). Primary stroke prevention worldwide: Translating evidence into action. *The Lancet Public Health*, *7*(1). 10.1016/S2468-2667(21)00230-9e74, 2022/01/01/. 10.1016/S2468-2667(21)00230-9 PMC8727355 34756176

[6] Hankey, G. J. (2003). Long-term outcome after ischaemic stroke/transient ischaemic attack, *Cerebrovascular Diseases*, *16*(1), 14–19. 10.1159/000069936. 10.1159/000069936 12698014

[7] Rössler, R., et al. (2020). Recovery of mobility function and life-space mobility after ischemic stroke: The MOBITEC-Stroke study protocol. *BMC Neurology*, *20*(1), 1–11. 10.1186/S12883-020-01920-Z/FIGURES/2. 2020/09/16. 32938425 10.1186/S12883-020-01920-Z/FIGURES/2 PMC7493846

[8] Park, J., & Kim, T. H. (2019, February 20). The effects of balance and gait function on quality of life of stroke patients, *NeuroRehabilitation*, *44*(1), 37–41. 10.3233/NRE-182467. 10.3233/NRE-182467 30741699

[9] Rowland, D. M., & Lewek, M. D. (2022). Linking gait mechanics with perceived quality of life and participation after stroke. *PLOS ONE*, *17*, 9. 10.1371/JOURNAL.PONE.0274511e0274511, 2022/09/01/. 10.1371/journal.pone.0274511 PMC9491527 36129881

[10] Deijle, I. A., Van Schaik, S. M., Van Wegen, E. E. H., Weinstein, H. C., & Kwakkel, G., & Van den Berg-Vos, R. M. (2017, January). Lifestyle interventions to prevent cardiovascular events after stroke and transient ischemic attack. *Stroke*, *48*(1), 174–179. 10.1161/STROKEAHA.116.013794. 10.1161/STROKEAHA.116.013794 27924055

[11] Goh, H. T., Nadarajah, M., Hamzah, N. B., Varadan, P., & Tan, M. P. (2016). Falls and fear of falling after stroke: A case-control study. *PM&R*, *8*(12), 1173–1180. 10.1016/j.pmrj.2016.05.012. 10.1016/j.pmrj.2016.05.012 27268565

[12] Cuccurullo, S. J., et al. (2022, May 1). Stroke recovery program with modified cardiac rehabilitation improves mortality, functional & cardiovascular performance. *Journal of Stroke and Cerebrovascular Diseases*, *31*(5), 106322. 35245825 10.1016/j.jstrokecerebrovasdis.2022.106322

[13] Buck, D., Jacoby, A., Massey, A., & Ford, G. (2000). Evaluation of measures used to assess quality of life after stroke. *Stroke*, *31*(8), 2004–2010. 10.1161/01.STR.31.8.2004. 10.1161/01.str.31.8.2004 10926971

[14] Vandenbroucke, J. P., et al. (2014, December). Strengthening the reporting of Observational studies in Epidemiology (STROBE): Explanation and elaboration, (in eng). *International Journal of Surgery*, *12*(12), 1500–1524. 10.1016/j.ijsu.2014.07.014. 10.1016/j.ijsu.2014.07.014 25046751

[15] Ewert, T., & Stucki, G. (2007). Validity of the SS-QOL in Germany and in survivors of hemorrhagic or ischemic stroke. *Neurorehabilitation and Neural Repair*, *21*(2), 161–168. 17312091 10.1177/1545968306292255

[16] Williams, L., Redmon, G., Saul, D. A., & Weinberger, M. (2001, January 1). Reliability and telephone validity of the stroke-specific quality of life (SS-QOL) scale, *Stroke*, *32*, 339–339. 10.1161/str.32.suppl_1.339-b.

[17] Lin, K., Fu, T., Wu, C., & Hsieh, C. (2011, January 19). Assessing the stroke-specific quality of life for outcome measurement in stroke rehabilitation: Minimal detectable change and clinically important difference, (in en). *Health Qual Life Outcomes*, *9*(1), 5. 10.1186/1477-7525-9-5. 10.1186/1477-7525-9-5 PMC3034658 21247433

[18] Lima, R. C. M., Teixeira-Salmela, L. F., Magalhães, L. C., & Gomes-Neto, M. (2008, April). Psychometric properties of the Brazilian version of the stroke specific quality of life scale: Application of the Rasch model, (in en). *Brazilian Journal of Physical Therapy*, *12*, 149–156. 10.1590/S1413-35552008000200012.

[19] Muus, I., Williams, L. S., & Ringsberg, K. C. (2007). Validation of the Stroke Specific Quality of Life Scale (SS-QOL): Test of reliability and validity of the Danish version (SS-QOL-DK), (in eng). *Clinical Rehabilitation*, *21*(7), 620–627. 10.1177/0269215507075504. 2007/07//. 17702704 10.1177/0269215507075504

[20] Montgomery, G. (2020, May 21). Determinants of performance in the timed up-and-go and six-minute walk tests in young and old healthy adults (in eng). *Journal of Clinical Medicine*, *9*(5), 1561. 10.3390/jcm9051561. 10.3390/jcm9051561 PMC7290512 32455757

[21] Bundesamt für Statistik (2018). *Die Schweizerische Gesundheitsbefragung 2017*.

[22] Tinetti, M. E., Richman, D., & Powell, L. (1990). Falls efficacy as a measure of fear of falling. *Journal of Gerontology*, *45*(6), P239-P243. 10.1093/geronj/45.6.P239. 10.1093/geronj/45.6.p239 2229948

[23] Yardley, L., Beyer, N., Hauer, K., Kempen, G., Piot-Ziegler, C., & Todd, C. (2005, November). Development and initial validation of the falls efficacy scale-international (FES-I) (in eng). *Age Ageing*, *34*(6), 614–619. 10.1093/ageing/afi196. 10.1093/ageing/afi196 16267188

[24] Brott, T. (1989, July). Measurements of acute cerebral infarction: A clinical examination scale, (in en). *Stroke*, *20*(7), 864–870. 10.1161/01.STR.20.7.864. 10.1161/01.str.20.7.864 2749846

[25] Lyden, P. D., Lu, M., Levine, S. R., Brott, T. G., & Broderick, J. (2001, June). A modified national institutes of health stroke scale for use in stroke clinical trials. *Stroke*, *32*(6), 1310–1317. 10.1161/01.STR.32.6.1310. 10.1161/01.str.32.6.1310 11387492

[26] Berger, K. (1999, February 1). [The reliability of stroke scales. The german version of NIHSS, ESS and Rankin scales], (in ger). *Fortschr Neurol Psychiatr*, *67*(2), 81–93. 10.1055/s-2007-993985. 10.1055/s-2007-993985 10093781

[27] van Swieten, J. C., Koudstaal, P. J., Visser, M. C., Schouten, H. J., & van Gijn, J. (1988, May). Interobserver agreement for the assessment of handicap in stroke patients (in eng). *Stroke*, *19*(5), 604-7. 10.1161/01.str.19.5.604. 10.1161/01.str.19.5.604 3363593

[28] Hinrichs, T. (2023 2023). Self-reported life-space mobility in the first year after ischemic stroke: Longitudinal findings from the MOBITEC-Stroke project. *J Neurol*, *270*(8), 3992–4003. 10.1007/s00415-023-11748-5. 10.1007/s00415-023-11748-5 PMC10157571 37140729

[29] Wong, G. K. C., et al. (2016). Clinically important difference of stroke-specific quality of Life Scale for aneurysmal subarachnoid hemorrhage. *Journal of Clinical Neuroscience*, *33*, 209–212. 10.1016/j.jocn.2016.05.029. 27460451 10.1016/j.jocn.2016.05.029

[30] Ekström, H., Dahlin-Ivanoff, S., & Elmståhl, S. (2011). Effects of walking speed and results of timed get-up-and-go tests on quality of life and social participation in elderly individuals with a history of osteoporosis-related fractures. *Journal of Aging and Health*, *23*(8), 1379–1399. 21868721 10.1177/0898264311418504

[31] Hirano, K., Imagama, S., Hasegawa, Y., Ito, Z., Muramoto, A., & Ishiguro, N. (2014). Impact of low back pain, knee pain, and timed up-and-go test on quality of life in community-living people. *Journal of Orthopaedic Science: Official Journal of the Japanese Orthopaedic Association*, *19*(1), 164–171. 2014/01/01/. 24132792 10.1007/s00776-013-0476-0

[32] Stegemöller, E. L., Nocera, J., Malaty, I., Shelley, M., Okun, M. S., & Hass, C. J. (2014). Timed up and go, cognitive, and Quality-of-life correlates in Parkinson’s Disease. *Archives of Physical Medicine and Rehabilitation*, *95*(4), 649–655. 10.1016/j.apmr.2013.10.031. 2014/04//. 24291596 10.1016/j.apmr.2013.10.031

[33] Ellis, T., Cavanaugh, J. T., Earhart, G. M., Ford, M. P., Foreman, K. B., & Dibble, L. E. (2011, November 1). Which measures of physical function and motor impairment best predict quality of life in Parkinson’s disease? *Parkinsonism & Related Disorders*, *17*(9), 693–697. 21820940 10.1016/j.parkreldis.2011.07.004 PMC3200468

[34] Stanghelle, B., Bentzen, H., Giangregorio, L., Pripp, A. H., & Bergland, A. (2019, November 4). Associations between health-related quality of life, physical function and pain in older women with osteoporosis and vertebral fracture. *BMC Geriatrics*, *19*(1), 298. 31684886 10.1186/s12877-019-1268-y PMC6829800

[35] Chen, C. M., Tsai, C. C., Chung, C. Y., Chen, C. L., Wu, K. P., & Chen, H. C. (2015, August 5). Potential predictors for health-related quality of life in stroke patients undergoing inpatient rehabilitation, (in eng). *Health and Quality of Life Outcomes*, *13*, 118. 10.1186/s12955-015-0314-5. 10.1186/s12955-015-0314-5 PMC4524441 26243294

[36] Grau-Pellicer, M., Chamarro-Lusar, A., & Medina-Casanovas, J., Serdà, B. C. (2019, July 4). Ferrer, Walking speed as a predictor of community mobility and quality of life after stroke. *26*, (5), 349–358. 10.1080/10749357.2019.1605751. 10.1080/10749357.2019.1605751 31063439

[37] Lee, K. B., Lim, S. H., Ko, E. H., Kim, Y. S., Lee, K. S., & Hwang, B. Y. (2015). Factors related to community ambulation in patients with chronic stroke. *Topics in Stroke Rehabilitation*, *22*(1), 63–71. 10.1179/1074935714Z.0000000001. 2015/02/01/. 25776122 10.1179/1074935714Z.0000000001

